# Targeted inhibition of RAGE in *substantia nigra* of rats blocks 6-OHDA–induced dopaminergic denervation

**DOI:** 10.1038/s41598-017-09257-3

**Published:** 2017-08-18

**Authors:** Juciano Gasparotto, Camila Tiefensee Ribeiro, Rafael Calixto Bortolin, Nauana Somensi, Thallita Kelly Rabelo, Alice Kunzler, Natália Cabral Souza, Matheus Augusto de Bittencourt Pasquali, José Claudio Fonseca Moreira, Daniel Pens Gelain

**Affiliations:** 1 Centro de Estudos em Estresse Oxidativo, Departamento de Bioquímica, Instituto de Ciências Básicas da Saúde, Universidade Federal do Rio Grande do Sul, Porto Alegre, RS Brazil; 2 Instituto de Medicina Tropical, Departamento de Bioquímica, Universidade Federal do Rio Grande do Norte, Natal, RN Brazil; 3 Unidade Acadêmica de Engenharia de Alimentos, Centro de Tecnologia e Recursos Naturais, Universidade Federal de Campina Grande – UFCG, Campina Grande, Paraíba Brazil

## Abstract

The receptor for advanced glycation endproducts (RAGE) is a pattern-recognition receptor associated with inflammation in most cell types. RAGE up-regulates the expression of proinflammatory mediators and its own expression via activation of NF-kB. Recent works have proposed a role for RAGE in Parkinson’s disease (PD). In this study, we used the multimodal blocker of RAGE FPS-ZM1, which has become available recently, to selectively inhibit RAGE in the substantia nigra (SN) of rats intracranially injected with 6-hydroxydopamine (6-OHDA). FPS-ZM1 (40 μg per rat), injected concomitantly with 6-OHDA (10 μg per rat) into the SN, inhibited the increase in RAGE, activation of ERK1/2, Src and nuclear translocation of NF-kB p65 subunit in the SN. RAGE inhibition blocked glial fibrillary acidic protein and Iba-1 upregulation as well as associated astrocyte and microglia activation. Circulating cytokines in serum and CSF were also decreased by FPS-ZM1 injection. The loss of tyrosine hydroxylase and NeuN-positive neurons was significantly inhibited by RAGE blocking. Finally, FPS-ZM1 attenuated locomotory and exploratory deficits induced by 6-OHDA. Our results demonstrate that RAGE is an essential component in the neuroinflammation and dopaminergic denervation induced by 6-OHDA in the SN. Selective inhibition of RAGE may offer perspectives for therapeutic approaches.

## Introduction

Parkinson’s disease (PD) is a progressive neurodegenerative disorder characterized by the specific loss of the nigrostriatal dopaminergic neurons, causing locomotor and postural deficits. Chronic neuroinflammation has been reported as a major contributor to PD^1^. The defense mechanisms in brain are able to protect against inflammatory processes. However, when inflammatory stressors accumulate beyond a threshold, which has not been defined until date, signaling pathways for neuronal death are triggered.

The amount of evidence linking RAGE to neurodegenerative diseases such as Alzheimer’s disease, PD, and Huntington’s disease has been increasing in the last few years^2^. RAGE belongs to the superfamily of immunoglobulins, which are present on the surface of many types of cells such as neurons, microglia, brain endothelial cells^3^ and astrocytes^4^. RAGE is a very promiscuous receptor binding many proteins such as S100b, HMGB1, HSP70, AGEs, β-amyloid and LPS among many others. New proteins with the capacity to bind RAGE are reported continuously.

Many animal models have been used to elucidate the mechanisms that trigger neurodegenerative diseases. We chose the rat model, where 6-hydroxydopamine (6-OHDA) is administrated unilaterally. This model has been studied extensively and the dopaminergic denervation in this rat model is similar to that in PD^5, 6^.

In the present work, we used a pharmacological antagonist, FPS-ZM1, for blocking RAGE in the SN to investigate potential neuroprotective effects against 6-OHDA-induced dopaminergic denervation. FPS-ZM1 is able to penetrate BBB and acts as a high affinity, multimodal blocker of RAGE through V domain-mediated ligand binding^3^. FPS-ZM1 treatment has been employed to study neurotoxicity models^7, 8^. FPS-ZM1 was injected into the same site of 6-OHDA. Proinflammatory, oxidative and neurotoxic effects of 6-OHDA in serum, CSF, and SN were evaluated. The results demonstrate a relationship between RAGE and 6-OHDA induced dopaminergic denervation in the SN. FPS-ZM1 was able to protect against most of the 6-OHDA-induced effects, suggesting that RAGE plays a pivotal role in the propagation/amplification of inflammatory effects and dopaminergic denervation consequent from 6-OHDA injection.

## Results

### RAGE is increased in the SN of 6-OHDA-treated rats

**Figure 1:**
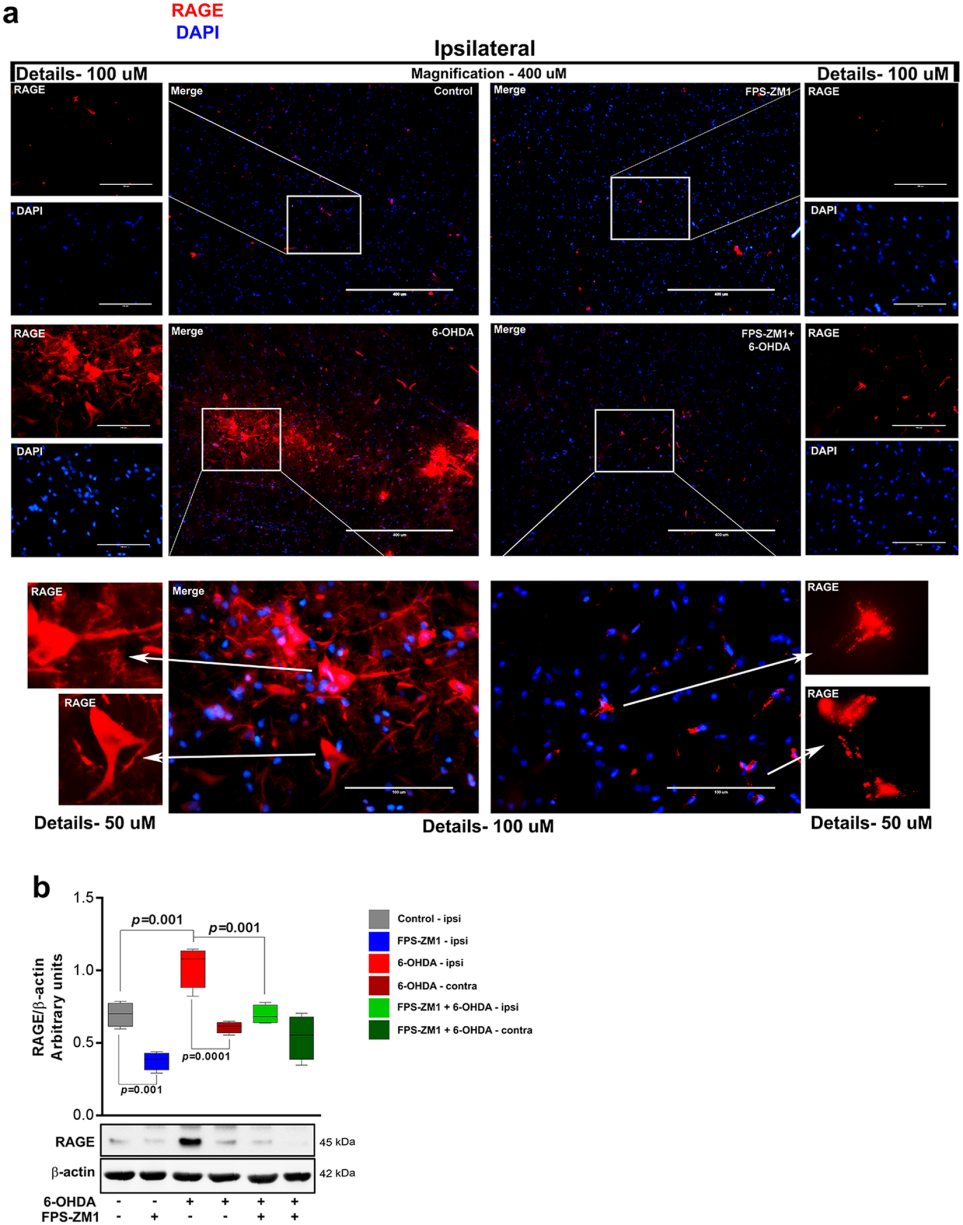
FPS-ZM1 blocked the increase in the levels of RAGE in rats administered with 6-OHDA. Rats were prepared for immunofluorescence and western blotting 15 days after the injection of 6-OHDA. (**a**) Representative immunofluorescence images of SN immunostained for RAGE and DAPI (*n* = 10 per group). The ipsilateral sides are shown. The microscopy images were taken with 400 μm of magnification and the squares represents the location of the approximation of 100 μm. (**b**) Representative western blots and quantification of RAGE (*n* = 6 per group). Each color in the graph represents a group and a brain location: gray - control/ipsilateral side; blue - FPS-ZM1/ipsilateral side; red - 6-OHDA/ipsilateral side; dark red - 6-OHDA/contralateral side; green - FPS-ZM1 + 6-OHDA/ipsilateral side; dark green - FPS-ZM1 + 6-OHDA/contralateral side. Values represent mean ± SD. One-way analysis of variance and Bonferroni Multiple Comparison *post-hoc* test were applied to all data. *p* values are embedded in the figure.

**Figure 2:**
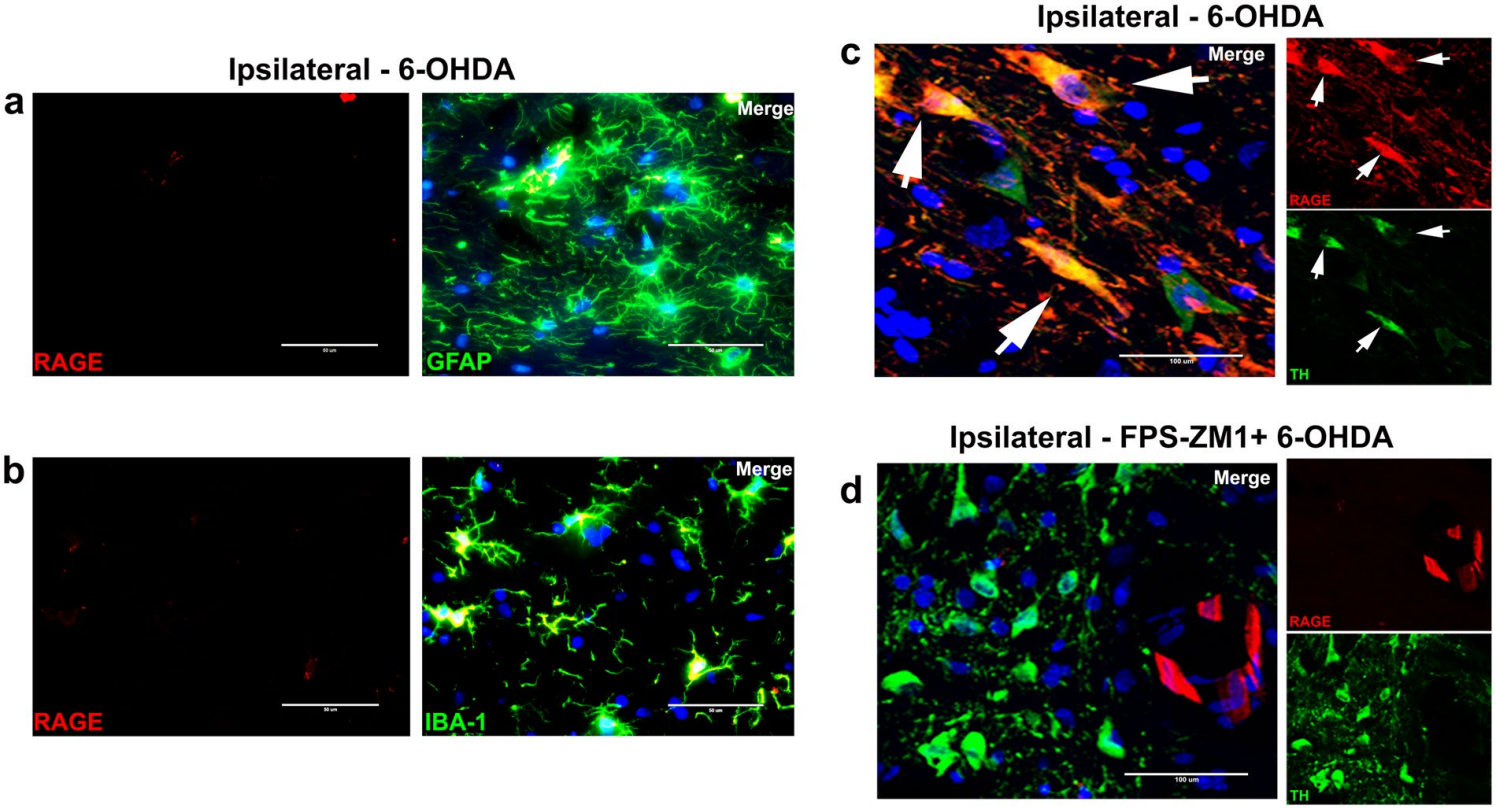
RAGE preferentially binds to dopaminergic neurons. Rats were prepared for immunofluorescence 15 days after 6-OHDA administration. The sections were co-immunostained for markers specific to different cell types and RAGE to evaluate the morphology and co-localization. (**a**) GFAP + RAGE, (**b**) IBA-1+ RAGE. The microscopy images were taken with 50 μm of magnification. (**c**) Confocal representative immunofluorescence images of SN co-immunostained for TH, RAGE and DAPI to ipsilateral 6-OHDA-induced and (**d**) ipsilateral FPS-ZM1 + 6-OHDA. The confocal microscopy images were taken with 100 μm of magnification and the z-axis layers are detailed in Sup. Fig. S1a and b. Representative immunofluorescence images of SN co-immunostained for different cell types (*n* = 10 per group): Only ipsilateral sides are showed.

The content of immunoreactive RAGE increased in the SN region of 6-OHDA-administered rats as shown by immunofluorescence analysis (Fig. 1a). Injection of RAGE blocking peptide FPS-ZM1 into the SN concomitantly with 6-OHDA inhibited the increase in RAGE content. Relative quantification by western blotting showed that RAGE increased by 50% in animals administered with 6-OHDA, which was inhibited by FPS-ZM1 by 46% (Fig. 1b). RAGE content and immunolocalization in the contralateral (not showed) SN was not affected, as expected (Fig. 1a). Interestingly, rats injected with FPS-ZM1 + 6-OHDA had increased RAGE localization in blood vessels as show detailed in Fig. 1a. In Control and FPZ-ZM1 groups, RAGE was not detected (Fig. 1a). To identify the cells in which RAGE was induced, we conducted co-immunostaining of RAGE with glial fibrillary acidic protein (GFAP - astrocyte marker), Iba-1 (microglial marker), or TH (dopaminergic neuron marker). The results showed the induction of RAGE mainly in TH+ cells, but not in astrocytes or microglia (Fig. 2a and b). Confocal microscopy scanning of Z-axis confirmed the co-localization of TH and RAGE staining in the same cells (Fig. 2c), demonstrating a different pattern of staining from rats treated with FPS-ZM1 and 6-OHDA, in which RAGE is typically localized in endothelial cells and absent in TH+ neurons (Fig. 2d). Details of Z-axis scanning are shown in supplementary figure S1a and b.

### NF-kB activation in SN of 6-OHDA-injected rats is mediated by RAGE

**Figure 3:**
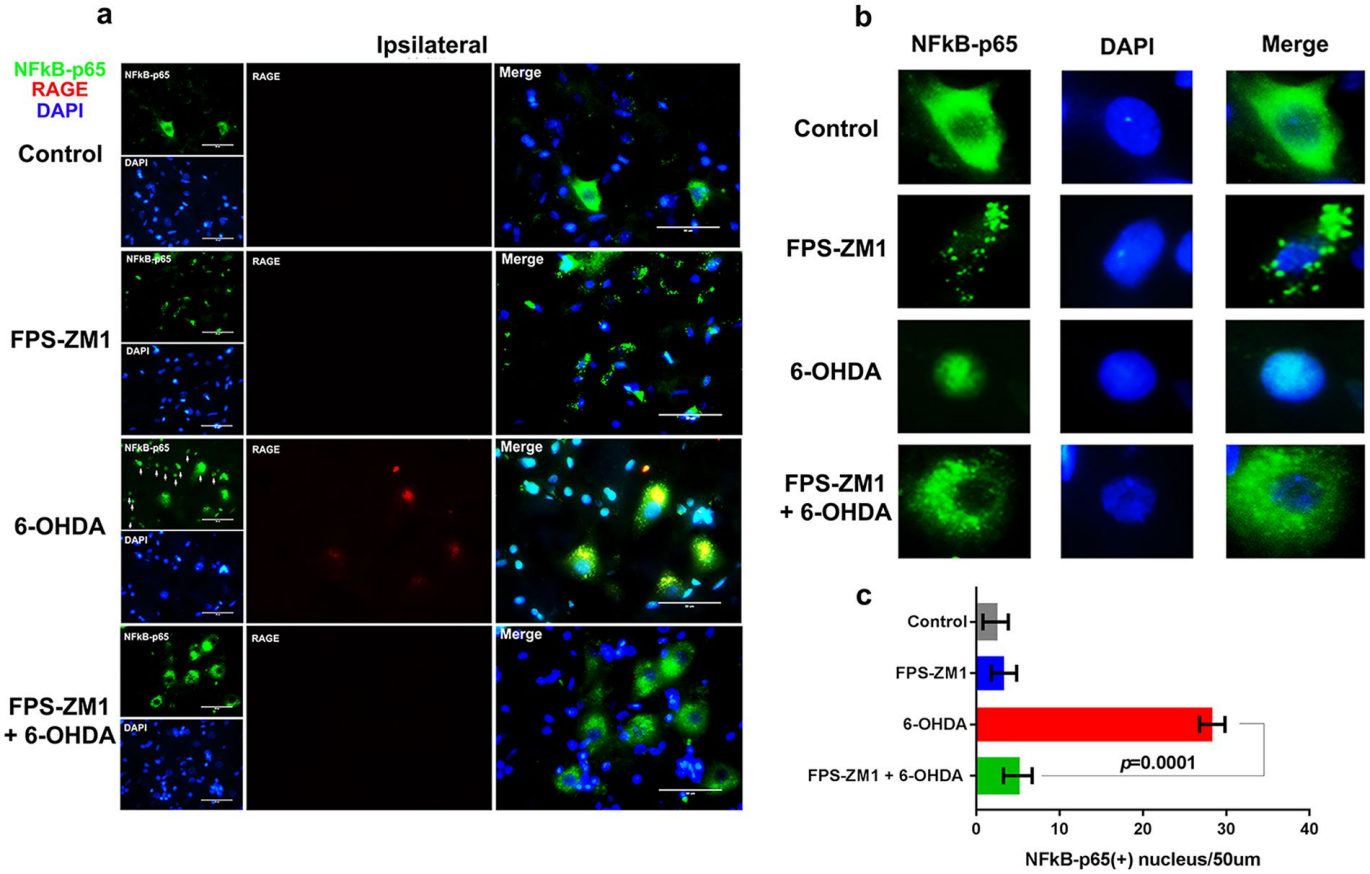
FPS-ZM1 blocked the 6-OHDA-induced nuclear translocation of NF-κB-p65. Rats were prepared for immunofluorescence 15 days after 6-OHDA administration. (**a**) Representative co-immunofluorescence images of SN immunostained for NF-κB-p65, RAGE and DAPI (*n* = 10 per group). The ipsilateral sides are showed. The microscopy images were taken with 50 μm of magnification. (**b**) Cell details. (**c**) p65+ nucleus quantification/picture with 50 μm magnification area. Values represent mean ± SD from 6 rats per group. One-way analysis of variance and Bonferroni Multiple Comparison *post-hoc* test were applied to all data. *p* values are embedded in the figure.

Following ligand binding, the intracellular machinery activated by RAGE induces the dissociation of p65 and p50 subunits from the inhibitory IkB subunit of the NF-kB complex in the cytoplasm, leading to p65 nuclear translocation. This results in transcriptional activation of proinflammatory genes and up-regulation of RAGE expression, as the RAGE gene (*AGER*) is also responsive to NF-κB transcriptional activity^9^. The immunofluorescence images of the transcriptional subunit, p65 of NF-κB show the translocation of p65 from cytoplasm to nucleus in many cells in 6-OHDA-induced group (Fig. 3a and detail in Fig. 3b). FPS-ZM1 completely blocked the 6-OHDA-induced translocation of p65 to nuclei. There were negligible number of p65+ nuclei in control group (2.3 ± 1.5) or animals receiving only FPS-ZM1 (3.4 ± 1.6) (Fig. 3c).

### ERK1/2 and Src activation in SN of 6-OHDA-injected rats are mediated by RAGE

**Figure 4:**
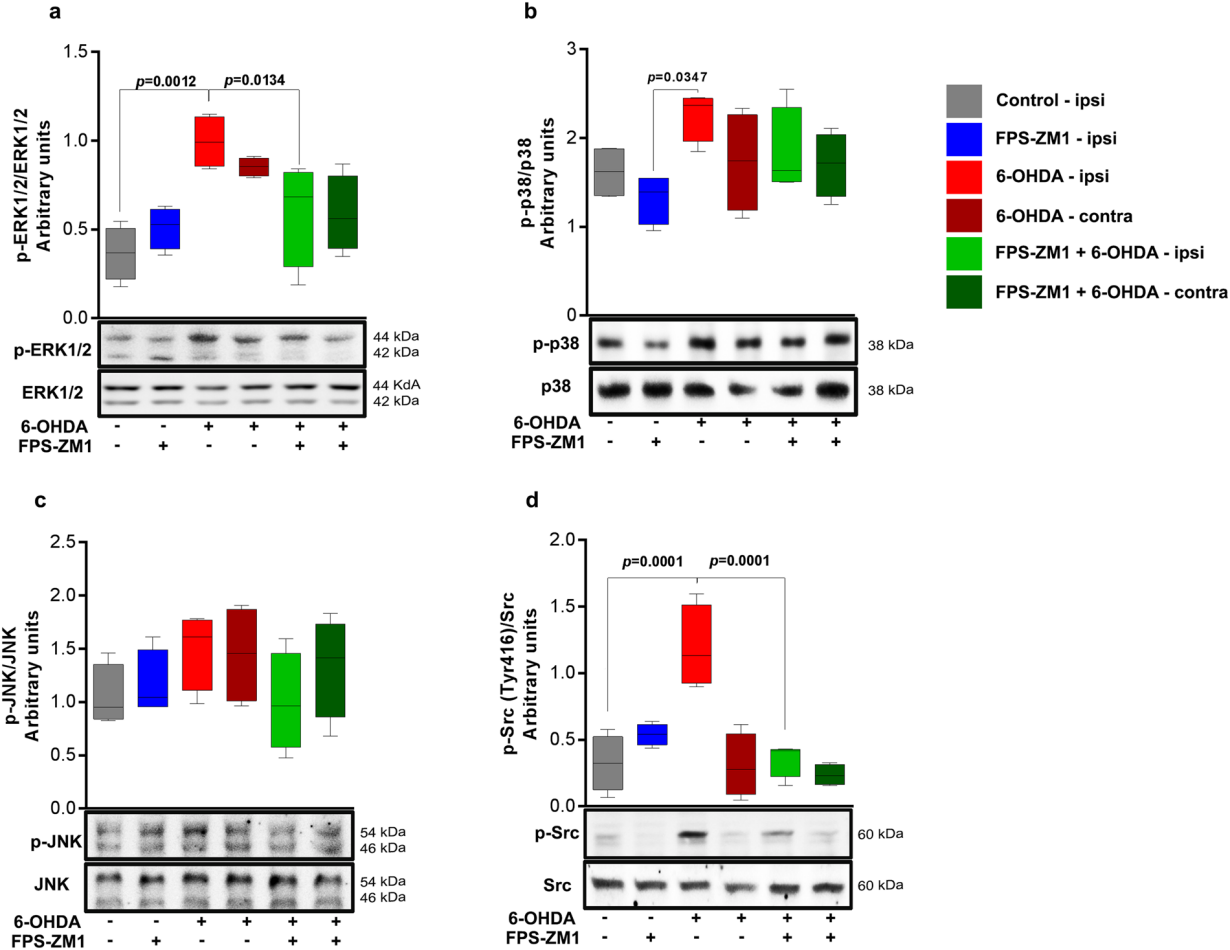
FPS-ZM1 inhibited 6-OHDA-induced MAPK-ERK 1/2 and Src phosphorylation. Tissue samples were prepared for western blotting assay 15 days after 6-OHDA-injection. (**a**) p-ERK 1/2. (**b**) p-p38. (**c**) p-JNK. (**d**) p-Src. The representative western blots of phosphorylated protein and total protein are shown below the graphs. Values represent mean ± SD from 6 rats per group. One-way analysis of variance and Bonferroni Multiple Comparison *post-hoc* test were applied to all data. *p* values are embedded in the figure.

RAGE ligand binding on cell membrane results in the activation of different protein phosphorylation cascades leading to the activation of transcription factors such as NF-kB^10^. We therefore examined the phosphorylation/activation of MAPKs, which are regulatory signaling molecules in inflammation and cell death and might be activated upon RAGE ligand binding. 6-OHDA induced the phosphorylation of ERK1/2 and Src (Fig. 4a and d), and the antagonist FPS-ZM1 suppressed ERK1/2 and Src activation. p38 was not inhibited to a significant extent by FPS-ZM1 and JNK phosphorylation was not significantly altered by 6-OHDA (Fig. 4b and c). These data suggest that ERK1/2 and Src phosphorylation in the SN of 6-OHDA-treated rats are evoked via a RAGE-dependent pathway.

### RAGE inhibition in SN blocks 6-OHDA-induced neuroinflammation

**Figure 5:**
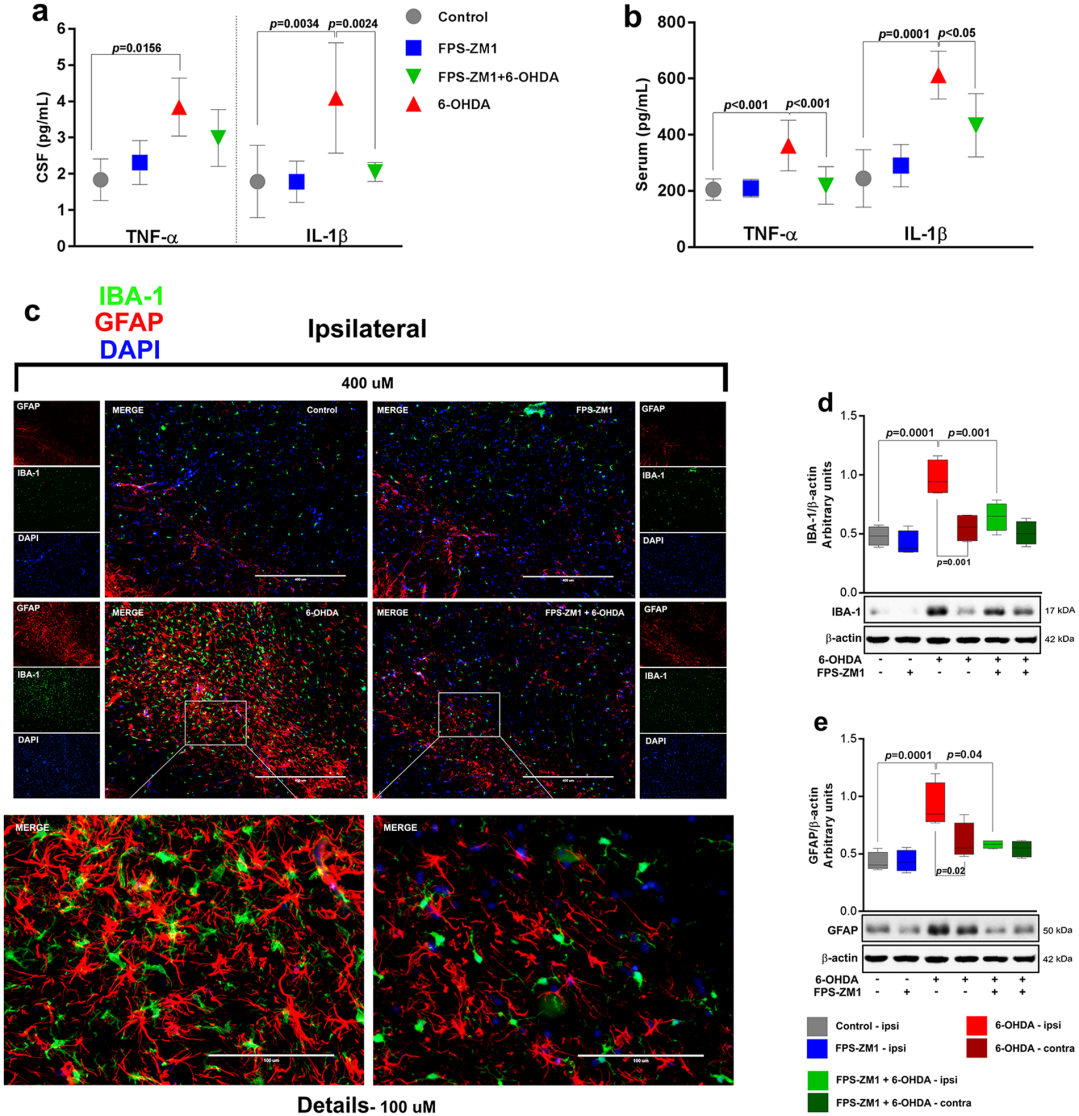
FPS-ZM1 protected rats from 6-OHDA-induced neuronal inflammation. CSF and serum were analyzed by ELISA assay 15 days after 6-OHDA administration. (**a**) Cerebrospinal fluid was analyzed for TNF-α and IL-1β. TNF-α and IL-1β levels are expressed in pg/mL. (**b**) Serum samples were analyzed for TNF-α and IL-1β. (**c**) Representative immunofluorescence images of SN co-immunostained for IBA-1, GFAP and DAPI (*n* = 10 per group). The ipsilateral sides are shown. The microscopy images were taken with 400 μm of magnification and the squares represents the location of the approximation of 100 μm. Representative western blots and quantification of (**d**) IBA-1 (*n* = 6 per group) and (**e**) GFAP (*n* = 6 per group). Each color in the graph represents a group and a brain location: gray - control/ipsilateral side; blue - FPS-ZM1/ipsilateral side; red - 6-OHDA/ipsilateral side; dark red - 6-OHDA/green - FPS-ZM1 + 6-OHDA/ipsilateral side; dark green - FPS-ZM1 + 6-OHDA/contralateral side. Values represent mean ± SD. One-way analysis of variance and Bonferroni Multiple Comparison *post-hoc* test were applied to all data. *p* values are embedded in the figure.

We also measured pro-inflammatory cytokines in CSF and serum (Fig. 5a and b). TNF-α and IL-1β were increased in CSF (110% and 130% respectively) and serum (70% and 150% respectively) of 6-OHDA-injected rats. FPS-ZM1 blocked the IL-1β increase in CSF (Fig. 5a), whereas both cytokines were blocked in serum (Fig. 5b). The administration of 6-OHDA triggers an inflammatory process that contributes to dopaminergic denervation. RAGE is a major regulator of chronic inflammation in several tissues, including CNS^11^. In order to assess the effect of RAGE inhibition on the inflammation of SN in 6-OHDA-injected rats, we evaluated the effect of RAGE inhibition on glial activation by assessing GFAP and Iba-1 immunostaining (Fig. 5c) and immunoblotting (Fig. 5d and e). The increases in the number of GFAP+ cells and the content of GFAP were inhibited by the presence of FSP-ZM1, indicating that RAGE inhibition decreases 6-OHDA-induced astrocyte activation (Fig. 5c and e). Similar effect was observed with Iba-1, suggesting the involvement of RAGE in 6-OHDA-induced microglia activation (Fig. 5c and d).

### RAGE inhibition in SN blocks 6-OHDA-induced dopaminergic denervation

**Figure 6:**
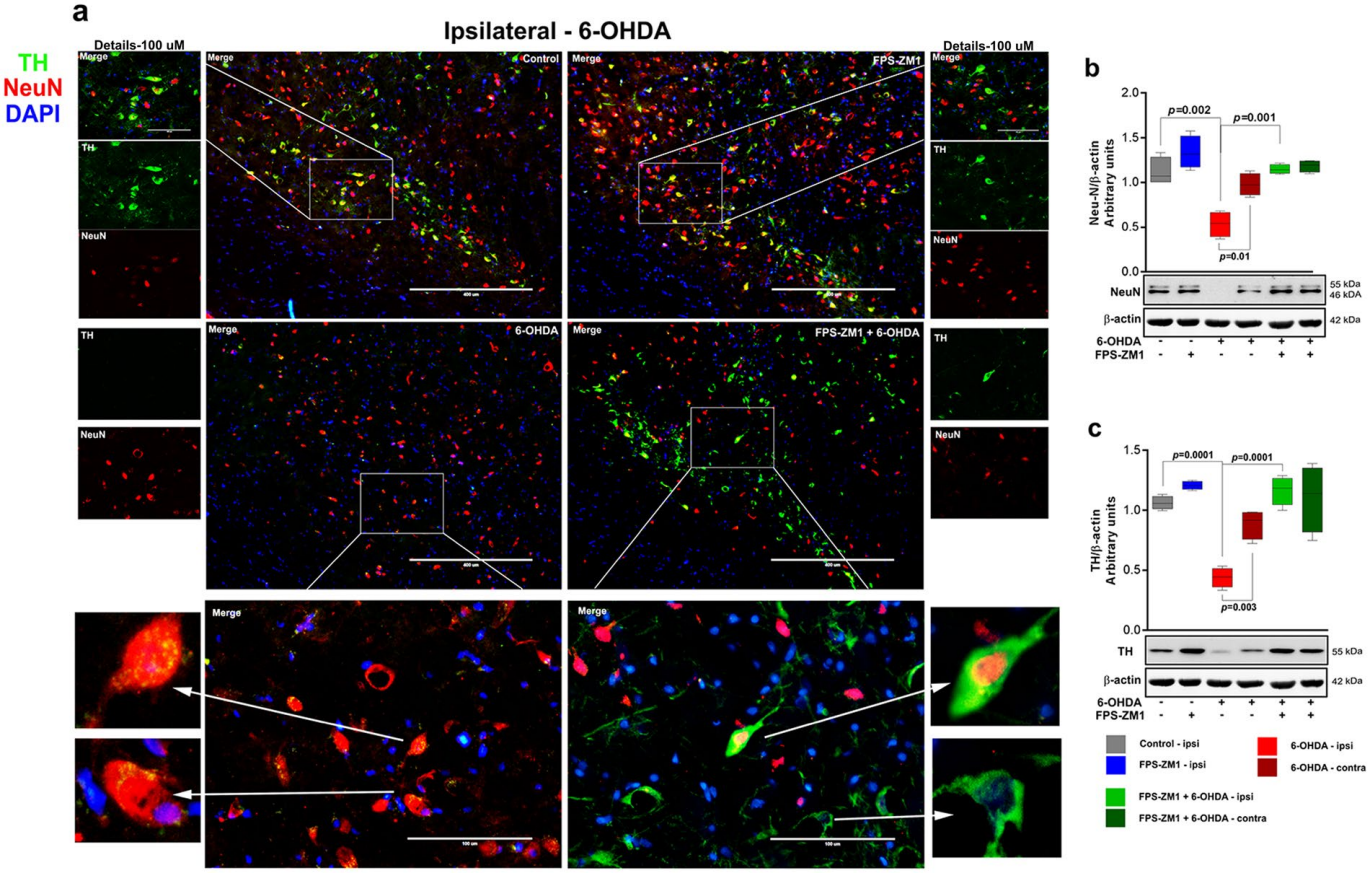
FPS-ZM1 inhibited 6-OHDA-induced neuronal death. Tissues were prepared for immunofluorescence and western blotting 15 days after 6-OHDA administration. (**a**) Representative immunofluorescence images of SN immunostained for TH, NeuN, and DAPI (*n* = 10 per group). The ipsilateral sides are shown. The microscopy images were taken with 400 μm of magnification and the squares represents the approximation to 100 μm. (**b**) Representative western blots and quantification of NeuN (*n* = 6 per group) and **c**) TH (*n* = 6 per group). Each color in the graph represents a group and a brain location: gray - control/ipsilateral side; blue - FPS-ZM1/ipsilateral side; red - 6-OHDA/ipsilateral side; dark red - 6-OHDA/contralateral side; green - FPS-ZM1 + 6-OHDA/ipsilateral side; dark green - FPS-ZM1 + 6-OHDA/contralateral side. Values represent mean ± SD. One-way analysis of variance and Bonferroni Multiple Comparison *post-hoc* test were applied to all data. *p* values are embedded in the figure.

Tyrosine hydroxylase (TH) is the rate-limiting enzyme in catecholamine synthesis and is the golden marker for dopaminergic neurons in the SN^12^. Death of dopaminergic neurons is a hallmark of PD. Therefore, we evaluated the effect of RAGE inhibition over dopaminergic neurons by immunostaining TH+ neurons and on other neurons by assessing a general neuronal marker, neuronal nuclear antigen (NeuN). The immunofluorescence analysis for neuronal markers revealed a significant loss of TH+ and NeuN+ cells in the SN of animals treated with 6-OHDA (Fig. 6a). Western blot analysis confirmed these observations (Fig. 6b and c). The RAGE inhibitor, FPS-ZM1 blocked the loss of TH and NeuN positive cells when administered concomitantly with 6-OHDA (Fig. 6a–c) and rescued the protein content. The blocking effect of RAGE inhibition in 6-OHDA-induced dopaminergic denervation is shown in nigrostriatal axis (Sup. Fig. S2). In addition, the TH-immunoreactivity images are shown in whole SN and striatum (caudate–putamen unit) (Sup. Fig. S3).

### RAGE inhibition in SN rescues locomotor, rotational and exploratory deficits induced by 6-OHDA

**Figure 7:**
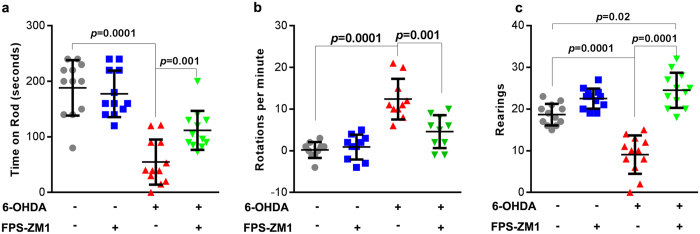
FPS-ZM1 protects rats from 6-OHDA-induced motor deficits. Locomotor abilities of rats injected with 6-OHDA were examined 14 days after the injection. (**a**) Rotarod test – total time on the rod limited to 4 min. (**b**) Rotational activity after apomorphine challenging. (**c**) Open field rearing test - duration limited to 5 min. Values represent mean ± SD from ten rats per group. One-way analysis of variance and Bonferroni Multiple Comparison *post-hoc* test were applied to all data. *p* values are embedded in the figure. *n* = 12 per group was used for rotarod and rearing test, *n* = 10 per group was used to measure the rotational activity.

Dopaminergic denervation induced by 6-OHDA is characterized by locomotor and exploratory deficits that can be assessed in behavioral tests. General motor performance was assessed 14 days after surgery, using the constant (21 RPM) rotarod test. Animals injected with 6-OHDA displayed significant reduction in the time on rotarod (60% of the control (Fig. 7a). Rats administered with both FPS-ZM1 and 6-OHDA presented an improved motor ability on rotarod test (10% higher than that of 6-OHDA group).

To evaluate the effectiveness and precision of the 6-OHDA injection the rotational behavior was assessed. The apomorphine-induced rotation is shown in Fig. 7b. The data demonstrate that 6-OHDA administration induced a rotational asymmetry, with rats displaying significant increase in continuous contralateral rotations following apomorphine injection (mean rates of 12 ± 1.5). Animals receiving FPS-ZM1 + 6-OHDA had significantly lower rotation rates (4.6 ± 1.2) induced by apomorphine injection. Control and FPS-ZM1 groups did not show any significant spontaneous rotation. The lesion severity of 6-OHDA model was established according to apomorphine-induced rotation^13^. The present model is characterized as severe, since rats exhibited more than 3 contralateral turns/minute. This magnitude of impairment is expected to display > 85% TH+ cell loss in SN and > 45% TH+ cell loss in VTA^13^.

We also evaluated the exploratory activity. Animals administered with FPS-ZM1 + 6-OHDA presented a higher exploratory activity in an open field test of rearing events, compared to animals receiving only 6-OHDA (Fig. 7c). Notably, 6-OHDA reduced the number of rearing episodes (50% of control), whereas rats exposed to FPS-ZM1 + 6-OHDA displayed a higher number of rearing episodes compared to the control group (Fig. 7c).

## Discussion

Although 6-OHDA-induced dopaminergic denervation has been a useful model largely utilized for understanding motor and biochemical dysfunctions associated to PD, it is not clear, whether dopaminergic cell death induced by this toxin shares common molecular mechanisms to those observed in patients. In this context, it must be considered that the 6-OHDA model does not replicate several pathological characteristics of PD, which poses limitations in relation to the interpretations of molecular mechanisms underlying the progression of dopaminergic cell death when using this model. For instance, animal models of 6-OHDA injection do not display Lewy bodies in the brain, a histopathological hallmark of PD^14, 15^. Besides, the dopaminergic denervation is localized and rapidly induced if compared to PD. In this disease, a progressive neuronal death evolves in the course of years and its consequence is not restricted to dopaminergic denervation, although this is the main cause of the characteristic motor symptoms^14^. Nonetheless, animal models of PD present different limitations regarding the multiple dysfunctions of the disease, and the 6-OHDA animal model is still considered a valid model to investigate behavioral and cellular features of PD^14^. Here, we performed a localized inhibition of RAGE with FPS-ZM1 (RAGE antagonist) to understand the involvement of RAGE in dopaminergic denervation and to test a potential strategy to prevent and neuronal death. RAGE interacts with a variety of ligands^4^ that may lead to inflammatory response causing brain injury and cell death^16^. To investigate the role of RAGE in SN, we administered 6-OHDA to induce neuronal death and co-administered FPS-ZM1 to inhibit the action of RAGE.

RAGE is a receptor with bad reputation^2^, fittingly so. RAGE is implicated in numerous neurodegenerative disorders such as Alzheimer’s disease, Parkinson’s disease, and Huntington’s disease^2^. Although there is ample evidence demonstrating the involvement of RAGE in neurodegenerative diseases^17^, the mechanism by which RAGE causes neuronal death remains unclear. Our results have demonstrated that 6-OHDA increased the number of RAGE-positive cells specifically in SN (Fig. 1a). Immunofluorescence visualization of a larger area in SN gives a general picture of the capacity of RAGE antibody to bind to various cell types. However, when we approximate the image magnification (Fig. 2c) it is possible to observe an accentuated presence of RAGE in dopaminergic neurons compared to that in astrocytes (Fig. 2a) and microglia (Fig. 2b).

Astrocytes are stellate-like cells and represent the most abundant cell type in most parts of the brain. The morphological changes induced in astrocytes by 6-OHDA (Sup. Fig. S1c) may result from changes of cytoskeletal proteins in microfilaments, intermediate filaments, and/or microtubules^18^. Microglia are highly dynamic cells, presenting a functional phenotype. Under both physiological and pathological conditions, they scan their environment and regulate tissue homeostasis^19^. In response to 6-OHDA lesion, microglia displayed a morphology associated to an activated phenotype (Sup. Fig. S1d) Furthermore, 6-OHDA reduced the number of TH+ neurons and induced characteristic morphological changes in surviving cells. The remaining TH+ cells showed a spherical format in comparison to the normal spindle forms presenting a well-designed axodendritic network (Sup. Fig. S1e and f). The neuroplasticity induced by 6-OHDA or derived toxins is unclear, however altered cell morphology may be an informative tool in documenting the changes caused by the neurotoxic insults to neuronal and nucleolar volume^20^.

NF-κB is a critical factor transducing a variety of inflammatory and pro- or anti-apoptotic signals in the cell, depending on the stimulus^21^. There was a massive presence of NF-κB-p65 in the nuclei of the SN cells of rats receiving 6-OHDA, indicating a transcriptional effect (Fig. 3b). NF-κB translocation also promotes the expression of proinflammatory cytokines^11^. The gene encoding RAGE contains functional binding elements for NF-κB. RAGE can also upregulate itself, perpetuating the neuroinflammation^21, 22^. FPS-ZM1 significantly suppresses the ERK1/2 phosphorylation and NF-κB translocation to the nuclei of the cells in the SN of rats injected with 6-OHDA (Fig. 3a–c). This strongly suggests that inhibiting RAGE activation blocked the signaling cascade and consequently blocked the inflammation and damage caused by 6-OHDA.

RAGE activation triggers MAPK-controlled phosphorylation cascades. Our results show that 6-OHDA significantly increased ERK1/2 and Src activation (Fig. 4a and d). ERK 1/2 is the main component of the MAPK pathway and when enhanced by prior Src phosphorylation, it plays a key role in RAGE signal, which may result in the upregulation of NF-κB^23^. In addition RAGE directly binds to ERK by a D-like domain, this interaction does not occur with others MAPKs as for p38 and JNK^23^. RAGE seems to have an important effect in this pathway, whereas FPS-ZM1 blocked the 6-OHDA-induced signaling otherwise activated by RAGE.

We observed an increase in TNF-α and IL-1β in 6-OHDA-induced rats, as demonstrated by the ELISA measurements in CSF and serum (Fig. 5a and b). The increases in these cytokines originate from many cells stimulated by 6-OHDA, mainly astrocytes and microglia, which overproduce and release TNF-α and IL-1β into CSF and serum. 6-OHDA generates a strong pro-inflammatory response and inflammatory condition in distinct cell types. Inhibiting RAGE with FPS-ZM1 blocked the release of cytokines (TNF-α in CSF was the exception) and the activation of astrocytes and microglia in SN (Fig. 5c–e). Taking these results together, RAGE seems to be a crucial factor that mediates the 6-OHDA-induced inflammatory process and contributes to neuronal degeneration.

Research with animal models and clinical studies suggested that RAGE^4^, protein S100B^24^ and HMGB1^25^ are potential contributors to the development of PD. These data are in accordance with our results and support our research focus to block RAGE, since inflammatory signaling and neuronal damage seem to occur through this receptor.

6-OHDA administration, as expected, reduced the number of TH and NeuN positive neurons (Fig. 6a–c). FPS-ZM1 protected against 6-OHDA-induced effects. These results provide strong evidence for the hypothesis that RAGE inhibition blocks all signaling cascades involved in neuroinflammation and dopaminergic denervation. The neuronal death in SN manifested in locomotor deficit was confirmed by rotarod test and apomorphine-induced rotations (Fig. 7a and b). In addition, the animals displayed depression-like behavior and decrease in exploratory interest (Fig. 7c) that is correlated with DA impairments^26^. FPS-ZM1 also protects against 6-OHDA-induced behavioral changes. There was an increase in exploratory interest, probably because there was an increase in the expression of neurotransmitters. The behavioral impairments along with reduced number of neurons in the SN provide concrete evidence for the progressive death of DA neurons. FPS-ZM1 protects against this effect of 6-OHDA.

## Conclusion

The multimodal blocker of RAGE, FPS-ZM1, shows a neuroprotective activity in the rat model of 6-OHDA-induced PD. FPS-ZM1 injected in the SN blocked dopaminergic denervation, neuroinflammation, and locomotor/exploratory deficits induced by 6-OHDA. The probable chain of events triggered by 6-OHDA in the SN of rats, according to our results, includes RAGE activation and upregulation, followed by activation of downstream signaling that involves ERK1/2 and Src, leading to p65 nuclear translocation and consequent pro-inflammatory response. This latter effect results in activation of astrocytes and microglia and increased circulating cytokines in CSF and serum. This, in turn, contributes to cell death and consequent loss of dopaminergic neurons (neurodegeneration), which is reflected in the deficits in locomotory and exploratory behavior. FPS-ZM1 was effective in blocking the dopaminergic denervation induced by 6-OHDA. However, it did not bring back the rotarod performance to control levels, indicating that molecular pathways other than RAGE also contribute to locomotor deficits caused by 6-OHDA, which is expected. Co-localization immunofluorescence results indicate that neurons are the main cells expressing RAGE in the SN of 6-OHDA-injected rats. Despite the limitations of this model, the present results indicate that the potential application of RAGE pharmacological inhibition in PD and the understanding of the role of this receptor in the pathophysiology of this disease must be investigated in more details.

## Methods

### Chemicals

Electrophoresis and immunoblotting apparatus were from Bio-Rad (Hercules, USA) and GE Healthcare Brazilian Headquarter (Sao Paulo, Brazil), respectively. The antibody information is listed in the descriptions of each technique. All other reagents used in this study were of analytical or HPLC grade.

### Ethics Statement

Our research protocol was approved under project number 27683 by the Ethical Committee for Animal Experimentation of the Universidade Federal do Rio Grande do Sul-Brazil (CEUA-UFRGS). All experimental procedures were performed in accordance with the guidelines of the National Institutes of Health^27^ and the Brazilian Society for Neuroscience and Behavior recommendations for animal care. All efforts were made to minimize animal suffering.

### Animals

Male Wistar rats (60 days old) bred in our facilities were maintained at constant temperature of 21 ± 1 °C and 12-hour light–dark cycle. They were caged in groups of four animals with free access (*ad libitum*) to water and standard commercial food (Chow Nuvilab CR-1 type; PR, Brazil). The rats were anesthetized with a single dose of ketamine (100 mg/kg; i.p.) and xilazine (10 mg/kg; i.p.) for all surgical procedures.

### FPS-ZM1 and 6-hydroxydopamine (6-OHDA) preparation

FPS-ZM1 (C_20_H_22_ClNO) is a non-toxic, tertiary amide compound that acts as a high affinity multimodal blocker of RAGE V domain-mediated ligand binding (K_i_ **= **25, 148, & 230 nM)^3^. It was dissolved in a small amount of dimethylsulfoxide (DMSO) and then diluted with saline to 10 μg/μL stock solution. The final DMSO concentration was 0.02%. FPS-ZM1 was purchased from Merck Millipore (MA, USA). Each rat received injection with 40 μg of FPS-ZM1 into the SN. 6-OHDA is a neurotoxic synthetic organic compound that destroys catecholaminergic terminals. It was prepared as a 5 μg/μL solution in 0.02% ascorbic acid dissolved in sterile saline, protected from heat and light. 6-OHDA was purchased from Sigma-Aldrich^**®**^ (MO, USA). Each rat was administered with 10 μg^28^ of 6-OHDA.

### Experimental design

The rats were randomly divided into four groups:

Group 1: control group received intranigral injection of saline solution.

Group 2: received FPS-ZM1 (intranigral injection).

Group 3: received 6-OHDA (intranigral injection).

Group 4: received FPS-ZM1 + 6-OHDA (intranigral injection).

Fourteen days after the induction of the SN lesion (see below), behavior tests were performed. On the 15^th^ day, all the animals were anaesthetized and CSF was sampled. Six animals from each group were decapitated and serum was collected, followed by dissection of the SN for analysis. Ten animals in each group were intracardially perfused for immunoflorescence assessment.

### Surgical Procedure

Anesthetized rats were immobilized by securing via ear and nose bars on a stereotaxic apparatus (Insight-EFF 338, SP, BRA). Fur was shaved with a pet clipper (SKU #: 09160-210 – Wahl; IL, USA) and 10% povidone-iodine solution was applied to sterilize the incision site. The skulls were trepanned with a dental drill (3 mm) at the appropriate location. A single dose (2 μL) of 6-OHDA^28^ or FPS-ZM1^3^ or saline was injected into the left SN at the following stereotaxic coordinates: antero-posterior (AP): −5.0 mm from bregma; medio-lateral (ML): ± 2.1 mm from the midline; dorso-ventral (DV): −8.0 mm from skull, according to the Rat Brain Atlas in Stereotaxic Coordinates Paxinos^29^, using a 10-μl Hamilton^®^ syringe 701SN, needle size 23s ga (Sigma-Aldrich^**®**^; MO, USA). Syringes were lowered into the brain at a rate of 2 mm/min. The chemicals were injected at a rate of 0.5 μL/min and the syringe was left in place for 2 min after injection, before drawing back at a rate of 2 mm/min. The incision was thoroughly cleaned with povidone-Iodine solution and closed using three sutures. Lactated Ringer’s solution (1 mL) was injected subcutaneously to replenish electrolytes. Nebacetin (Medley; RS, BRA) was applied topically to the incision to prevent infections. The animals were removed from stereotaxic frame and placed in a controlled temperature recovery cage (37 °C) until they regained consciousness. The animals were returned to the housing facility 2 h after the surgery.

### Immunofluorescence microscopy

Fifteen days after the surgery, rats were perfused via the vascular system with descending aorta clamped. Sterile saline was administered for 10 min followed by perfusion with 4% paraformaldehyde (PFA) solution in PBS, pH 7.4, for 10 more minutes. The brains were then carefully recovered and maintained in 4% PFA for 24 h at 4 °C. The brains were then transferred into 15% sucrose solution for 24 h at 4 °C followed by immersion in 30% sucrose for additional 24 h at 4 °C. Brains were lightly dried and frozen at −20 °C. After 24 h, the SN region was sectioned in to slices of 15 μm thickness on the coronal plane using a cryostat at −20 °C (Jung Histoslide 2000R; Leica; Heidelberg, Germany). A total of 20–30 slices per rat containing SN were collected in PBS containing 0.1% triton ×100. The free-floating sections were incubated in 5% albumin for 2 h to block nonspecific binding. The sections were then incubated with antibodies for 48 h at 4 °C. The details of the antibody source and dilutions are as follows. Anti-GFAP (1:500- G6171) and DAPI for nucleic acid staining (1:500; D9542) were from Sigma-Aldrich^**®**^ (MO, USA). Anti-IBA-1 (1:500; 019-19741) was from Wako Chemicals USA, Inc. (VA, USA). Anti-RAGE (1:200; PA1-84173) was from Thermo Fisher Scientific (MA, USA). Anti-NeuN (1:500; MAB377) was from Merck Millipore (MA, USA). Anti-NF-κB -p65 (1:200; 6956) and Anti-TH (1:200; 2792 S) were from Cell Signaling Technology^**®**^ (MA, USA). Antibodies were diluted in PBS containing 2% bovine serum albumin. Primary antibodies were excluded from the incubation of negative controls. After washing four times with 0.1% PBS, tissue sections were incubated with secondary antibodies, which included anti-rabbit Alexa 488 or 555; anti-mouse Alexa 488 or 555 and Alexa anti-goat 555 from Cell Signaling Technology^**®**^ (MA, USA), all of them diluted 1:500 in PBS containing 2% BSA. After incubation in secondary antibodies for 1 h at room temperature (21 ± 3 °C), the sections were washed several times in 0.1% PBS, transferred to gelatinized slides, mounted with FluorSave™ (345789- Merck Millipore; MA, USA) and covered with coverslips. The images were acquired using a Microscopy EVOS^®^ FL Auto Imaging System (AMAFD1000 - Thermo Fisher Scientific; MA, USA). The Z-axis images were collected using a Laser-scanning confocal microscopy (Olympus FV 1000, Tokyo, Japan).

### Enzyme-linked immunosorbent assay (ELISA) determination of cytokines

TNF-α (RAB0479-1KT) and IL-1β (RAB0272-1KT) were quantified with commercial kits from Sigma-Aldrich^**®**^ (MO, USA). The CSF from *cisterna magna* and blood serum samples were incubated in ELISA plates and processed further according to the manufacturer’s protocol.

### Western blotting

For immunoblotting experiments, tissues were prepared using a radioimmunoprecipitation assay buffer protocol^30^. Total proteins (30 μg/well) were fractionated by SDS-PAGE and electroblotted onto nitrocellulose membranes with Trans-Blot Semi-Dry Electrophoretic Transfer Cell (Bio-Rad; CA, USA). Protein loading and electroblotting efficiency were verified through Ponceau S staining. After washing with TTBS (100 mM Tris – HCl, pH 7.5, containing 0.9% NaCl, and 0.1% Tween-20), the membranes were incubated with primary antibodies (1:500 dilutions) for 20 min at room temperature in SNAP i.d. 2.0 Protein Detection System (Merck Millipore; MA, USA). The membranes were washed again with TTBS. Polyclonal and monoclonal antibodies used were from Cell Signaling Technology^**®**^ (MA, USA) and included the following: anti-p-ERK-44/42 (Thr202/Tyr204) (9101), anti-ERK-44/42 (9102), anti-p-Src (Tyr416) (2101), anti-Src (2108), p-p38 (4511), anti-p38 (8690), anti-p-SAPK/JNK (Thr183/Tyr185) (9255), and anti-SAPK/JNK (9252). The blots were incubated with anti-rabbit, goat or mouse peroxidase-linked secondary antibody for an additional 20 min in SNAP (1:5000 dilution) and washed again. The immunoreactivity was detected by enhanced chemiluminescence using Supersignal West Pico Chemiluminescent kit from Thermo Fisher Scientific (MA, USA). The chemiluminescence was captured with an ImageQuant LAS 4000 (GE Healthcare; SP, Brazil). Densitometric analysis of the images were performed using ImageJ software (ImageJ v1.49, National Institute of Health, USA). Blots were developed to be linear in the range used for densitometry. All results were expressed as relative ratio to β-actin (A1978) from Sigma-Aldrich^**®**^ (MO, USA) or total protein content.

### Behavioral tests (locomotor, rotational and exploratory activities)

The motor system was evaluated using the rotarod test. The protocol was performed at a constant speed of 21 rpm. The cut-off time was 240 s^31^. The animals were acclimated to the apparatus with three prior training sessions at one-hour intervals. The duration on rotarod during experimental sessions was measured. The mean of 3 attempts were used for the statistical analyses.

Apomorphine-induced rotation test was performed to study the hypersensitivity of the lesioned SN. Apomorphine 0.1 mg/kg (dissolved in a 0.2 mg/mL ascorbic acid in 0.9% saline solution) was subcutaneously injected and tested over a 40 min session. Animals were allowed to habituate for 5 min after injection before the recording of rotations began in cylinder rotameter (400 mm diameter). Full body ipsilateral and contralateral side rotations were counted by an observer who was blind to the animal pretreatments. The data were expressed as the net (contralateral - ipsilateral turns) average rotations per min (RPM)^32^. Apomorphine was purchased from Sigma-Aldrich^**®**^; MO, USA.

The other parameter evaluated was the number of rearing events. In an open field test, the rearing episodes were scored when the animal displayed a vertical exploratory activity^33^.

### Protein assay

Total protein was quantified by Bradford assay and used to normalize all data^34^.

### Statistical analysis

Statistical analysis was performed using GraphPad Prism version 7 (GraphPad Software Inc., CA, USA). Data were evaluated by one-way ANOVA followed by Bonferroni multiple comparison *post-hoc* test. The results are expressed as mean ± SD. Differences were considered significant when *p* < 0.05.

## Electronic supplementary material

Supplementary figures
