# Supplementary material for: Targeted inhibition of RAGE in substantia nigra of rats blocks 6-OHDA–induced dopaminergic denervation
Source: Sci Rep. 2017 Aug 18;7:8795. doi: 10.1038/s41598-017-09257-3 (PMC5562811; doi:10.1038/s41598-017-09257-3)
Supplement: Supplementary file 1 — Supplementary figures [file 41598_2017_9257_MOESM1_ESM.pdf]

Targeted inhibition of RAGE in *substantia nigra* of rats blocks 6-OHDA–induced dopaminergic denervation

Juciano Gasparotto[a*], Camila Tiefensee Ribeiro[a], Rafael Calixto Bortolin[a], Nauana Somensi[a], Thallita Kelly Rabelo[a] Alice Kunzler[a], Natália Cabral Souza[b], Matheus Augusto de Bittencourt Pasquali [c], José Claudio Fonseca Moreira[a], Daniel Pens Gelain[a]

[a] Centro de Estudos em Estresse Oxidativo, Departamento de Bioquímica, Instituto de Ciências Básicas da Saúde, Universidade Federal do Rio Grande do Sul – Porto Alegre, RS – Brazil;

[b] Instituto de Medicina Tropical, Departamento de Bioquímica, Universidade Federal do Rio Grande do Norte – Natal, RN – Brazil;

[c] Unidade Acadêmica de Engenharia de Alimentos, Centro de Tecnologia e Recursos Naturais, Universidade Federal de Campina Grande – UFCG, Campina Grande - Paraíba - Brasil

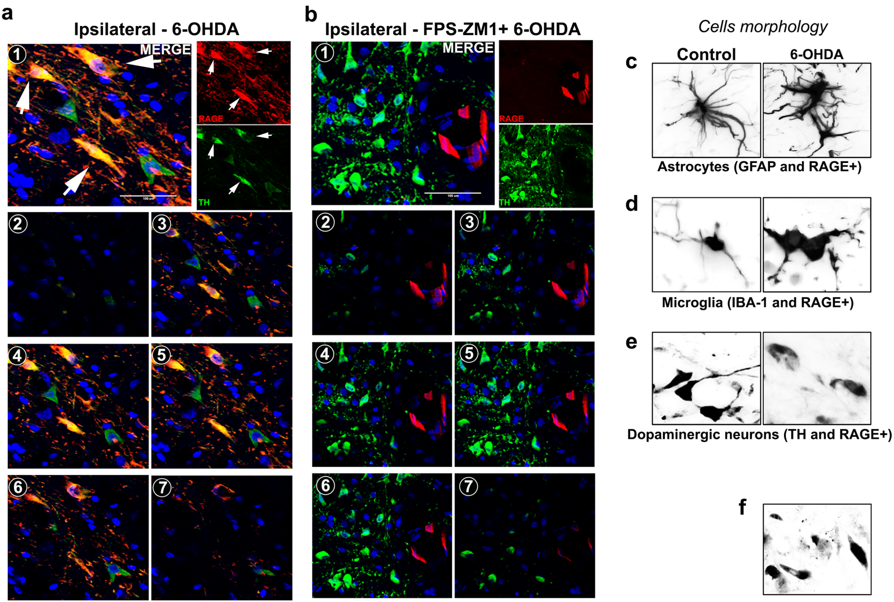

Supplementary figure S1. RAGE is localized to TH(+) neurons.

SN were prepared for immunofluorescence 15 days after the administration of 6-OHDA.

a) Confocal representative immunofluorescence images of SN co-immunostained for TH, RAGE and DAPI to ipsilateral 6-OHDA-induced and **b**) ipsilateral FPS-ZM1+6-OHDA (n=10 per group): Only ipsilateral sides are showed.

The confocal microscopy images were taken with 100 um of magnification and the z-axis layers (1-7) are detailed. Detailed cell morphology focused in one cell and shown in black and white: **c**) Astrocytes. **d**) Microglia. **e**) Dopaminergic neurons. **f**) Cells immunostained only with RAGE.

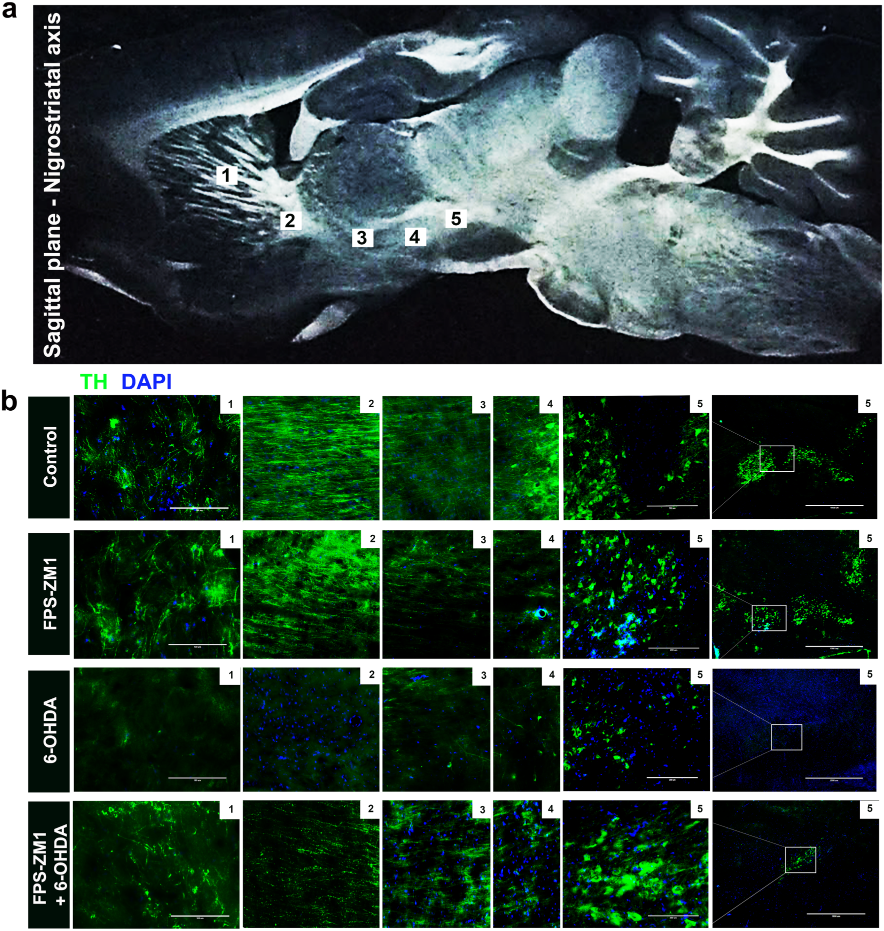

Supplementary figure S2. FPS-ZM1 blocked denervation 6-OHDA-induced in nigrostriatal axis
Rats were prepared for immunofluorescence 15 days after the injection of 6-OHDA.
a) Picture of rat brain, the numbers (1-5) represents the location of the image microscopy (100 or 400 um of magnification). **b)** The extent of damage to the nigrostriatal axis immunostained for TH and DAPI
The ipsilateral sides are shown (*n*=3 per group).

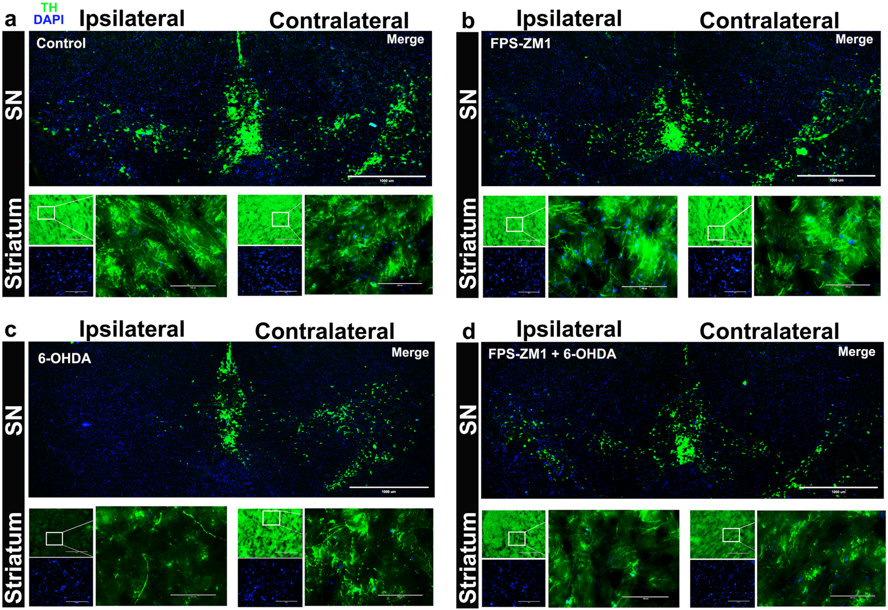

Supplementary figure S3. FPS-ZM1 blocked denervation 6-OHDA-induced in SN and striatum
SN and striatum were prepared for immunofluorescence 15 days after the injection of 6-OHDA. **a)** control, **b)** FPS-ZM1, **c)** 6-OHDA and **d)** FPS-ZM1+6-OHDA representative immunofluorescence images of SN and striatum co-immunostained for TH and DAPI (n=10 per group). The ipsilateral and contralateral sides are shown. The microscopies magnification is detail in each image.
